# Substance P Differentially Modulates Firing Rate of Solitary Complex (SC) Neurons from Control and Chronic Hypoxia-Adapted Adult Rats

**DOI:** 10.1371/journal.pone.0088161

**Published:** 2014-02-07

**Authors:** Nicole L. Nichols, Frank L. Powell, Jay B. Dean, Robert W. Putnam

**Affiliations:** 1 Department of Neuroscience, Cell Biology and Physiology, Wright State University Boonshoft School of Medicine, Dayton, Ohio, United States of America; 2 Division of Physiology, University of California San Diego School of Medicine, La Jolla, California, United States of America; 3 Department of Molecular Pharmacology and Physiology, University of South Florida Morsani College of Medicine, Tampa, Florida, United States of America; INRA, France

## Abstract

NK1 receptors, which bind substance P, are present in the majority of brainstem regions that contain CO_2_/H^+^-sensitive neurons that play a role in central chemosensitivity. However, the effect of substance P on the chemosensitive response of neurons from these regions has not been studied. Hypoxia increases substance P release from peripheral afferents that terminate in the caudal nucleus tractus solitarius (NTS). Here we studied the effect of substance P on the chemosensitive responses of solitary complex (SC: NTS and dorsal motor nucleus) neurons from control and chronic hypoxia-adapted (CHx) adult rats. We simultaneously measured intracellular pH and electrical responses to hypercapnic acidosis in SC neurons from control and CHx adult rats using the blind whole cell patch clamp technique and fluorescence imaging microscopy. Substance P significantly increased the basal firing rate in SC neurons from control and CHx rats, although the increase was smaller in CHx rats. However, substance P did not affect the chemosensitive response of SC neurons from either group of rats. In conclusion, we found that substance P plays a role in modulating the basal firing rate of SC neurons but the magnitude of the effect is smaller for SC neurons from CHx adult rats, implying that NK1 receptors may be down regulated in CHx adult rats. Substance P does not appear to play a role in modulating the firing rate response to hypercapnic acidosis of SC neurons from either control or CHx adult rats.

## Introduction

The neuropeptide substance P is involved in several physiological processes including cardiovascular, respiratory, gastrointestinal, and nociceptive processes and even modulation of the immune response [1], [2], [3], [4]. Substance P has been shown to affect ventilation as well. When substance P was applied to the nucleus tractus solitaries (NTS), the minute ventilation increased [5], [6], [7], [8], [9]. Further, when saporin conjugated to substance P (which will lesion neurons expressing NK1 receptors) was microinjected into different chemoreceptor sites, including the retrotrapezoid nucleus (RTN) and medullary raphé, the hypercapnic ventilatory response (both frequency and tidal volume) was decreased [10], [11], [12]. This suggests that neurons expressing neurokinin receptor 1 (NK1) may play a role in modulating central chemosensitivity. Further, substance P has been shown to affect the hypoxic ventilatory response, where injections of an NK-1 receptor antagonist into the ventricles of the brain decreased the hypoxic ventilatory response [13].

Substance P is known to be stored and released from carotid body afferent neurons located in the proximal petrosal ganglion and jugular ganglion [14]. These peripheral afferents have their first primary synapse in the caudal portion of the NTS [14], [15], [16], [17], [18], [19], a known site for central chemosensitivity [20, 21, 22, 23. 24]. However, it is not known if the afferents synapse on caudal NTS neurons that are responsive to hypercapnia.

The primary receptor that substance P binds to is the NK1 receptor. NK1 receptors have been found in various regions involved in central chemosensitivity [10], [25]. Thus, NK1 receptors have been proposed as markers of chemosensitive neurons [25]. NK1 receptors are susceptible to desensitization due to receptor down regulation with prolonged exposure to substance P [26], [27], and have been found to be susceptible to internalization in the NTS after conditions such as exercise [28]. One such condition that could cause down regulation of NK1 receptors is a chronic exposure to hypoxia. Chronic hypoxia causes increased release of substance P from the peripheral afferents that leave the carotid body [29], a site known to contain cells sensitive to hypoxia, implying that there will be increased substance P release onto caudal NTS neurons [30], [31]. It has been shown that increased binding of substance P to NK1 receptors can cause desensitization both *in vitro* and *in vivo* [26], [32], [33]. Furthermore, receptor down regulation due to desensitization could lead to decreased excitatory effects of substance P on respiration. Lastly, exposure to chronic intermittent hypoxia causes a decrease in NK1 receptor density on NTS neurons [34]. However, it has not been shown how chronic intermittent hypoxia affects the chemosensitive response of NTS neurons or whether chronic hypoxia affects NK1 receptor density on NTS neurons.

We have reported that chronic hypoxia causes a suppression of the chemosensitive response of NTS neurons from adult rats (observed as an increase in the percentage of NTS neurons inhibited by hypercapnia) [35]. We wanted to know if desensitization of the response to substance P [26] could play a role in the suppression of the chemosensitive response we observe in SC neurons after chronic hypoxia. Therefore, the main goal of this study was to examine the role of substance P in the chemosensitive response of SC neurons from control and chronic hypoxia-adapted (CHx) rats. Substance P was shown to modulate basal firing rate of SC neurons from control and CHx adult rats, but it had no effect on the firing rate response of SC neurons to hypercapnic acidosis.

A preliminary account of some of these data has previously been published [36].

## Experimental Procedures

### Ethics Statement

All procedures involving animals were approved by the Institutional Animal Care and Use Committee at Wright State University and were in agreement with standards set forth in the National Institutes of Health Guide for Care and Use of Laboratory Animals. All efforts were made to minimize animal suffering. Wright State University is accredited by AAALAC and is covered by NIH Assurance (no. A3632-01).

### Chronic Adaptation to Hypoxia

Chronic adaptation to hypoxia was studied as described previously [35]. Briefly, 4–6 Sprague-Dawley male adult rats, of approximate age P60, were placed together in a plexiglas chamber (cylinder 91.4 cm long and 27.9 cm in diameter) in which they were exposed to a continuous flow of air at approximately 0.5 atmospheres absolute (ATA)_,_ which is approximately 0.1 ATA of O_2_ (chronic hypoxia) for ≥7 days, until each animal was euthanized and tested. These adult rats, which have been adapted to chronic hypoxia, will be referred to as CHx rats. This protocol had chamber controls, where 4–6 male adult rats were placed together in the chamber with a continuous flow of room air (21% O_2_, ∼0.21 ATA). All rats were allowed food and water *ad libitum* in the Plexiglas chamber. To assess the effectiveness of chronic hypoxia, hematocrit and body weight were both recorded for each rat as previously reported [35]. As previously observed [35], [37], hematocrit increased and body weight decreased in CHx rats. The total number of rats used in this study was 48 rats; 24 rats were used for chronic hypoxia studies and 24 rats were used for chamber controls. Temperature and relative humidity were recorded for all groups on HOBO HO8 RH recorders (Onset Computer Corporation, Bourne, MA) that were placed in the Plexiglas chamber. The range of temperatures (17–23°C) and relative humidity (23–60%) in the chamber were similar to those in a previous study [35].

### Solutions

Artificial cerebral spinal fluid (aCSF) contained the following (in mM): 124 NaCl, 5.0 KCl, 2.4 CaCl_2_, 1.3 MgSO_4_, 1.24 KH_2_PO_4_, 26 NaHCO_3_, and 10 glucose, and was equilibrated with 95% O_2_/5% CO_2_ (pH∼7.45 at 37°C) [23], [24], [35], [38], [39], [40]. Synaptic blockade (SNB) solution (high Mg^2+^, low Ca^2+^) was modified from aCSF in order to block chemical synapses [21] where CaCl_2_ and MgSO_4_ were adjusted to 0.2 mM and 11.4 mM, respectively. The whole cell patch intracellular solution contained (in mM): 130 K^+^-gluconate, 10 K^+^-HEPES, 0.4 EGTA, 1 MgCl_2_, 0.3 Na_2_GTP, and 2 Na_2_ATP, (pH = 7.45 at room temperature) [23], [24], [35], [38], [39], [40]. For the measurement of intracellular pH (pH_i_), 1 mM of the pH-sensitive fluorescent dye 8-hydroxypyrene-1,3,6-trisulfonic acid, trisodium salt (HPTS, pyranine) (Invitrogen, Eugene, OR) was added to the whole cell patch intracellular solution. Substance P methyl ester (5 mg) (American Peptide, Sunnyvale, CA and Sigma, St. Louis, MO) stock solution was prepared in 3.7 mL of 100 mM acetic acid and then aliquots of 50 µL were stored at −20°C until needed. 50 µL of substance P stock solution was directly added to 50 mL of SNB solution to give a final concentration of 1 µM substance P for experiments. A stock solution of L-703,606 oxalate salt (5 mg; NK1 receptor antagonist) (Alexis Biochemicals, San Diego, CA and Sigma, St. Louis, MO) was prepared in 8.35 mL of ddH_2_0 and then aliquots of 50 µL were stored at 4°C until needed. 50 µL of L-703,606 stock solution was directly added to 50 mL of SNB solution to give a final concentration of 1 µM L-703,606 for experiments. All chemicals were purchased from Sigma (St. Louis, MO) except where noted.

### Slice Preparation

Slices for study were prepared from control and CHx adult male rats (P57–P64) as previously described (35) Briefly, adult male rats were anesthetized with a brief exposure to CO_2_ (100%) until unresponsive, and then rapidly decapitated [24], [35]. The brainstem was removed and submerged in aCSF equilibrated with 5% CO_2_/95% O_2_ gas mixture. Transverse slices (300 µm) were prepared on a vibratome (Pelco 101, series 1000) beginning at the obex and extending rostrally for ∼1 mm and were allowed to recover for at least 1 hour at room temperature in aCSF equilibrated with a 5% CO_2_/95% O_2_ gas mixture. Individual slices for study from control and CHx adult male rats were placed in a superfusion chamber on the stage of an upright Nikon Optiphot-2 microscope. Slices were immobilized with a nylon grid and superfused at ∼2–4 ml/min with aCSF equilibrated with a 5% CO_2_/95% O_2_ gas mixture at 37°C.

Individual neurons from SC slices were studied in SNB solution. The basic experimental protocol for studies consisted of a 5 minute exposure to SNB equilibrated with 5% CO_2_/95% O_2_, a 10–15 minute exposure to hypercapnic (15% CO_2_/85% O_2_) solution followed by a 5–10 minute washout in the original solution (hypercapnic test of chemosensitive response). This protocol was then repeated in the same neuron with 1 µM substance P. For studies involving L-703,606 a 5 minute pre-exposure to the antagonist was used followed by both the antagonist and substance P in 5% CO_2_ or in hypercapnic solution.

### Imaging of Fluorescent Neurons

pH_i_ was measured as previously described [24], [39], [40], [41]. Individual SC neurons were loaded with 1 mM of the pH-sensitive dye, pyranine, through a whole cell patch pipette. It was previously shown that it takes ∼5–10 minutes to achieve stable intracellular fluorescence [41]. Fluorescence images were collected every minute at 515 nm emission with alternating 450 and 410 nm excitation using a Sutter Lambda 10-2 filter wheel. Images were processed with MetaFluor 7.1.4.0 software (Molecular Devices) to yield R_fl_. We used the equation (pH = 7.4969+log (N_fl_-0.2003)/(2.0194-N_fl_); r^2^ = 0.99), derived from a calibration curve that was generated previously for pyranine loaded into SC neurons from adult rats [24], to convert N_fl_ into pH_i_.

### Electrophysiological Studies

The blind whole-cell patch clamp technique was used to measure neuronal membrane potential (V_m_) and integrated firing rate as described previously [24], [35], [42]. The experimental setup that was used has been previously described [23], [24], [35], [43], [44], [45]. Briefly, whole cell patch pipettes were used [24], [35] and moved into the superfusion solution and down to the slice. Positive pressure was applied and then taken off once tip impedance resulted in a 1–2 mV downward deflection. Negative pressure was applied to the pipette to obtain a giga-ohm seal, brief suction was applied to the pipette to rupture the membrane, and then V_m_ and integrated firing rate were measured throughout the experiment. Integrated firing rate (Hz) was determined as previously described [24], [35] and analyzed using pClamp 8.2 software. Viable neurons had a stable V_m_ of between −40 and −60 mV and fired action potentials that crossed through zero. The firing rate response to hypercapnia was quantified as previously described using two measures: percentage of neurons activated by acute hypercapnia, and the magnitude of the firing rate response to acute hypercapnia, calculated as the chemosensitivity index (CI) according to the equation of Wang and Richerson [46]. A neuron was designated as CO_2_-activated if its CI was greater than 120% or CO_2_-inhibited if its CI was less than 80%.

### Statistical Analysis

A 2-way ANOVA with a repeated measure design was used to compare: 1) basal firing rate of SC neurons from control and CHx rats in the absence and presence of substance P; 2) basal firing rate in the absence and presence of substance P of SC neurons that were activated, inhibited or unchanged in response to hypercapnic acidosis from control rats; 3) basal firing rate in the absence and presence of substance P of SC neurons that were activated, inhibited or unchanged in response to hypercapnic acidosis from CHx rats; 4) the adapted firing rate response to substance P for the initial, peak and final firing rate of SC neurons from control and CHx rats; 5) the plateau firing rate response to substance P for the initial and plateau firing rate of SC neurons from control and CHx rats; 6) basal pH_i_ in the absence and presence of substance P of SC neurons from control and CHx rats; 7) basal pH_i_ in the absence and presence of substance P of SC neurons that were activated, inhibited or unchanged in response to hypercapnic acidosis from control rats; 8) basal pH_i_ in the absence and presence of substance P of SC neurons that were activated, inhibited or unchanged in response to hypercapnic acidosis from CHx rats; and 9) pH_i_ in response to hypercapnic acidosis in the absence and presence of substance P of SC neurons from control and CHx rats. A one-way ANOVA was used to compare chemosensitvity indices of SC neurons that responded to hypercapnia. If significant differences existed following ANOVAs, then multiple comparisons were done using Tukey’s or Fisher’s least significant difference *post hoc* tests with levels of significance of P<0.05 (SigmaPlot version 12.0; Systat Software Inc., San Jose, CA, USA). Fisher’s exact tests were used to compare differences between the percentages of SC neurons that responded to hypercapnia. Paired t-tests were used to compare differences between the change of firing rate and pH_i_ induced by substance P of SC neurons from control and CHx rats, and the change of firing rate induced by L-703,606 of SC neurons from control and CHx rats. All differences between groups were considered significant if P<0.05 and all values were expressed as means ±1 S.E.M.

## Results

### Effect of Substance P on Basal Firing Rate of SC Neurons from both Control and CHx Rats

**Figure 1:**
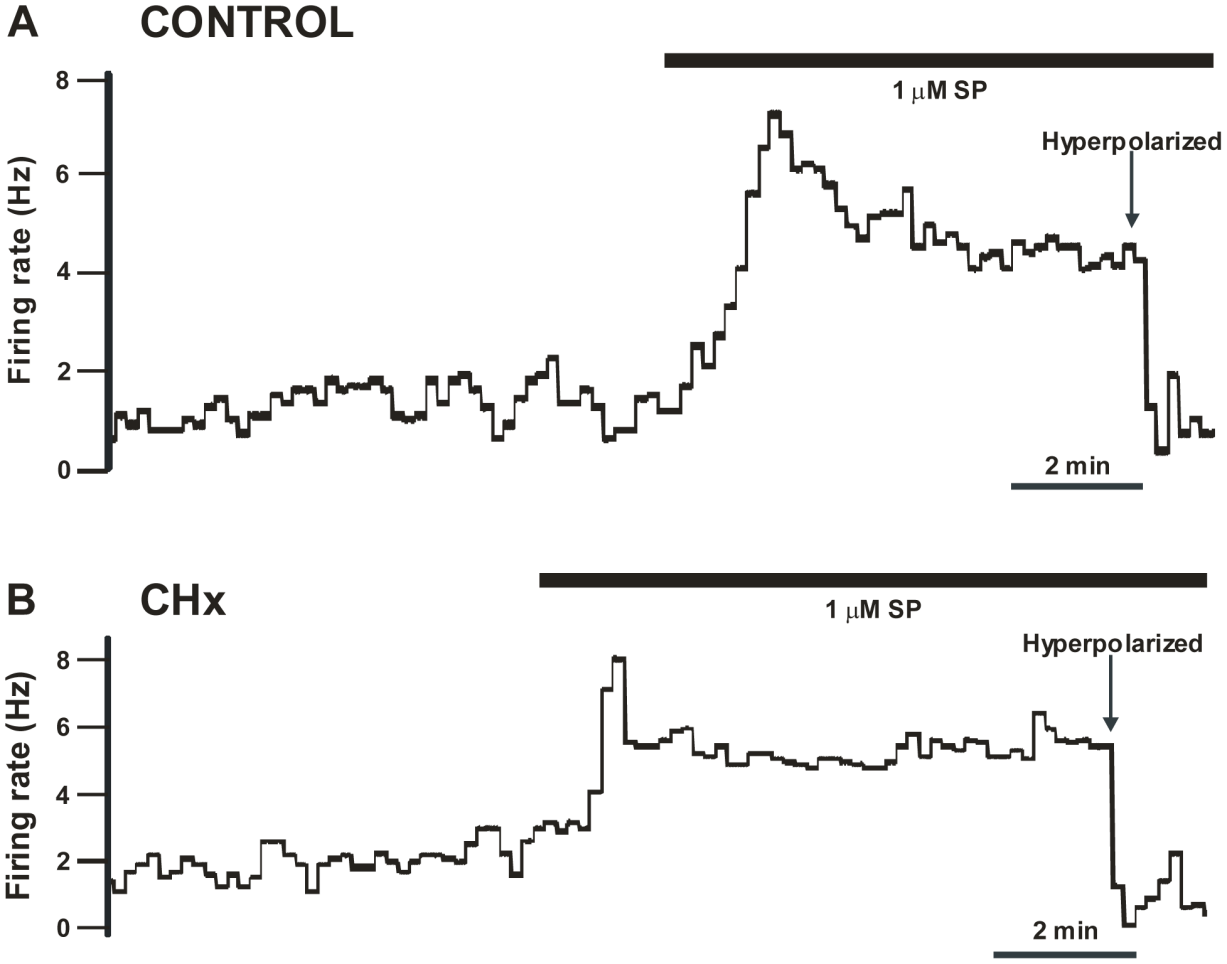
Adapted basal firing rate response following substance P. Representative samples of adapted basal firing rate responses to prolonged substance P (SP) exposure of SC neurons from both a control (A) and a CHx rat (B). Black bar represents SP exposure. (A) Sample adapted response to SP of an SC neuron from a control rat. Notice that basal firing rate first peaked in response to SP, but then firing rate adapted down to around 4–5 Hz. Before we exposed the neuron to hypercapnia, we hyperpolarized the neuron by injecting negative DC current to return firing rate back to its initial values. (B) Sample adapted response to SP of an SC neuron from a CHx rat. Notice that basal firing rate first peaked in response to SP, but then firing rate adapted down to approximately 4–5 Hz. Before we exposed the neuron to hypercapnia, we first hyperpolarized the neuron by injecting negative DC current to return firing rate back to initial values. Note, that in both types of rats, firing rate adapted to ∼4–5 Hz.

**Figure 2:**
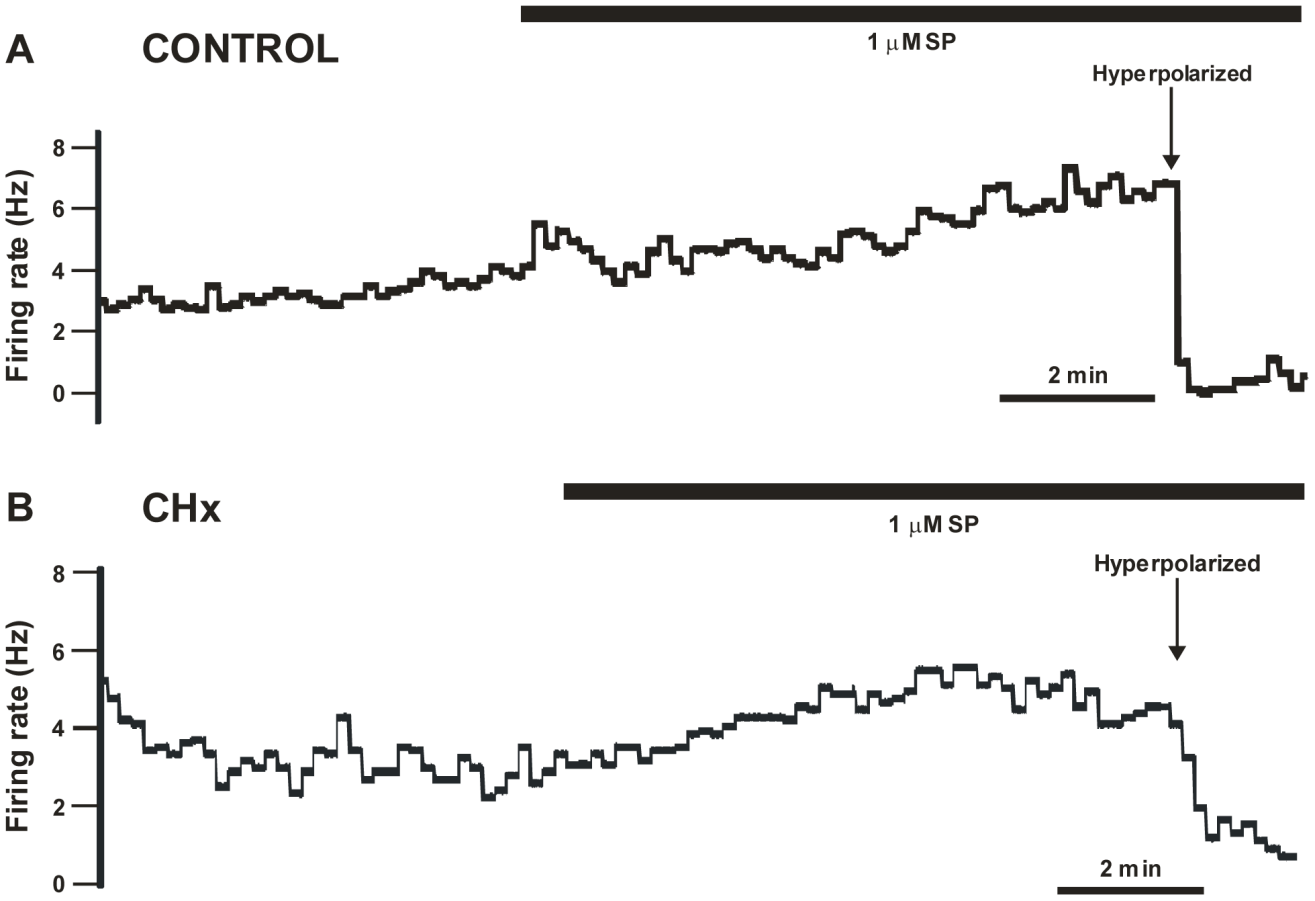
Plateau basal firing rate response following substance P. Representative samples of plateau basal firing rate responses to prolonged substance P (SP) exposure of SC neurons from both a control (A) and a CHx rat (B). Black bar represents SP exposure. (A) Sample plateau response to SP of an SC neuron from a control rat. Notice that basal firing rate slowly rose in response to SP and plateaued around 4–5 Hz. Before we exposed the neuron to hypercapnia, we first hyperpolarized the neuron by injecting negative DC current to return firing rate back to initial values. (B) Sample plateau response to SP of an SC neuron from a CHx rat. Notice that basal firing rate plateaued in response to SP to approximately 4–5 Hz. Before we exposed the neuron to hypercapnia, we first hyperpolarized the neuron by injecting negative DC current to return firing rate back to initial values. Note, that in both types of rats, firing rate plateaued at ∼4–5 Hz.

We studied the effects of substance P on the basal firing rate of SC neurons by exposing the neuron to the neuropeptide until firing rate stabilized (≥8–15 minutes). All SC neurons tested increased firing rate in response to substance P. We observed two types of increased firing rate responses to substance P: a response that first peaked and then adapted (termed the adapted response) (Fig. 1); and a response that slowly plateaued (termed the plateau response) (Fig. 2). These two types of responses were seen in SC neurons from both control rats (Fig. 1A for adapted responses and Fig. 2A for plateau responses) and CHx rats (Fig. 1B for adapted responses and Fig. 2B for plateau responses). After the firing rate stabilized in the presence of substance P, we hyperpolarized the V_m_ to bring firing rate back towards its initial value in the presence of substance P (see Figs. 1 and 2 for examples). This was done to allow measures of the chemosensitive response in the presence of substance P compared to the absence of substance P to be performed from the same initial firing rates.

To investigate if there were differences in the substance P response between control and CHx rats, we first quantified the maximal response induced by substance P. We combined responses from adapted and plateau response neruons (N = 22 for control rats and N = 23 for CHx rats). In SC neurons from control rats, substance P significantly increased firing rate from 1.74±0.29 Hz to 6.45±0.75 Hz (P<0.001) and SC neurons from CHx rats had a basal firing rate that increased significantly from 2.11±0.28 to 4.78±0.51 Hz in response to substance P (P<0.001; data not shown). However, SC neurons from CHx rats had a significantly smaller change in firing rate in response to substance P when compared to that seen in control rats (change of 4.71±0.59 Hz for control rats and 2.67±0.59 Hz for CHx rats; P<0.05; data not shown). It is possible that the smaller response to substance P in SC neurons from CHx rats may be due to decreased NK1 receptor expression, as observed in rats exposed to chronic intermittent hypoxia [34].

**Figure 3:**
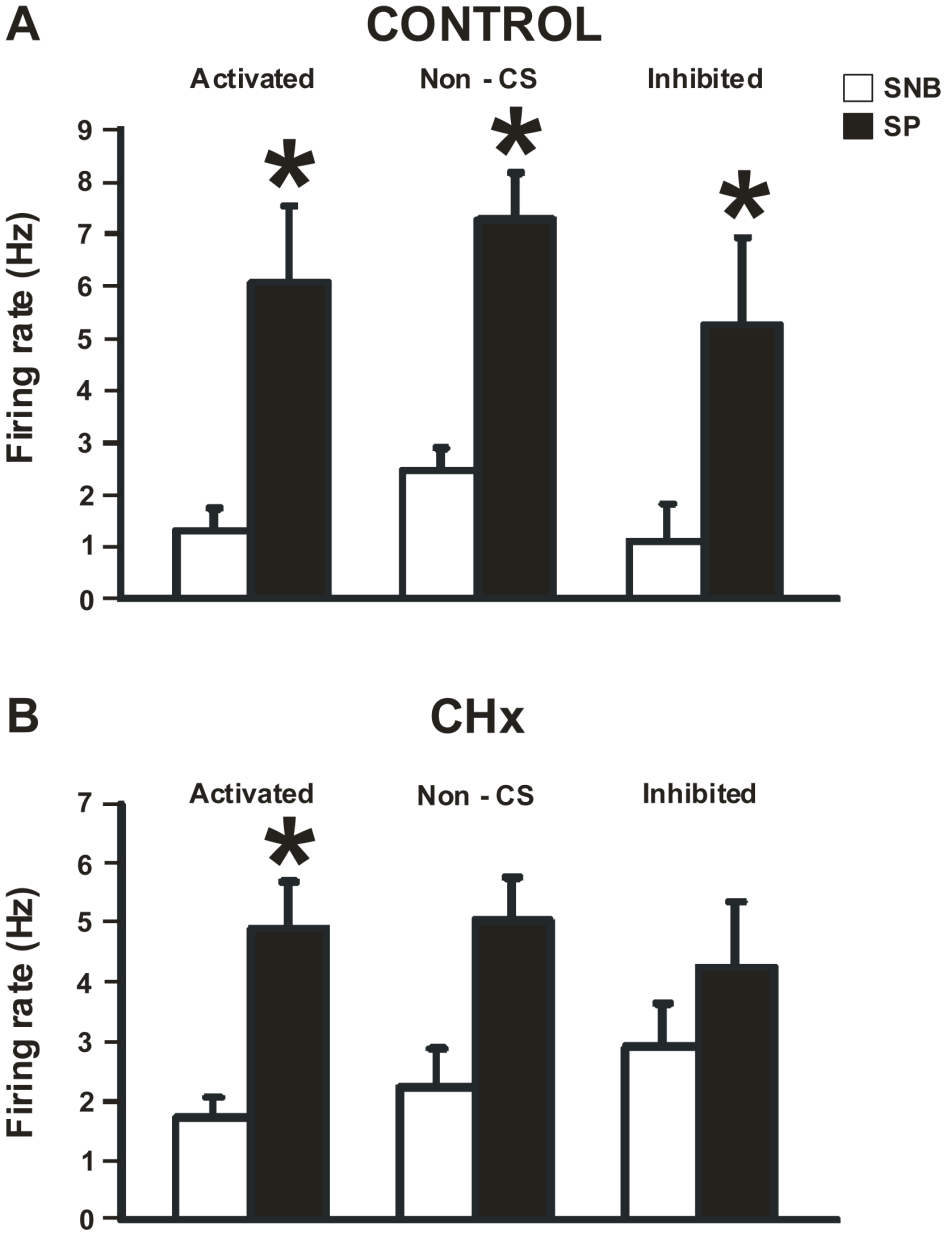
Average basal firing rate response to substance P of all SC neurons. The average basal firing rate response to substance P (SP) for neurons that were CO_2_-activated, non-chemosensitive (non-CS), or CO_2_-inhibited in response to hypercapnia in SC neurons from both control (A) and CHx rats (B) where white bars are in the absence of SP and black bars are in the presence of SP. (A) SP significantly increased the basal firing rate of CO_2_-activated (N = 10; P<0.001), non-CS (N = 9; P<0.001) and CO_2_-inhibited SC neurons (N = 3; P<0.05) from control rats. There were no differences between CO_2_-activated, non-CS or CO_2_-inhibited SC neurons when comparing firing rate in the absence or the presence of SP. (B) SP significantly increased the basal firing rate of CO_2_-activated SC neurons (N = 13; P<0.001), but not non-CS (N = 4) or CO_2_-inhibited (N = 6) SC neurons from CHx rats. There were no differences between CO_2_-activated, non-CS or CO_2_-inhibited SC neurons when comparing firing rate in the absence or the presence of SP. * indicates firing rate in SP is significantly different from firing rate in SNB. In all cases, the height of a bar represents the mean firing rate ± S.E.M.

We next addressed whether the substance P response of SC neurons depended on their response to hypercapnia. SC neurons were classified as activated, non-chemosensitive or inhibited, based on their firing rate response to hypercapnic acidosis, and then the maximal basal firing rate response induced by substance P between the three groups were compared (Fig. 3). In control rats, substance P significantly (P<0.001) increased firing rate in SC neurons regardless of their response to hypercapnia (Fig. 3A). However, in CHx rats, substance P only increased firing rate significantly (P<0.001) in CO_2_-activated SC neurons, but not in non-chemosensitive or CO_2_-inhibited SC neurons (Fig. 3B). Thus, it appears that SC neurons from CHx rats have a smaller response to substance P.

**Figure 4:**
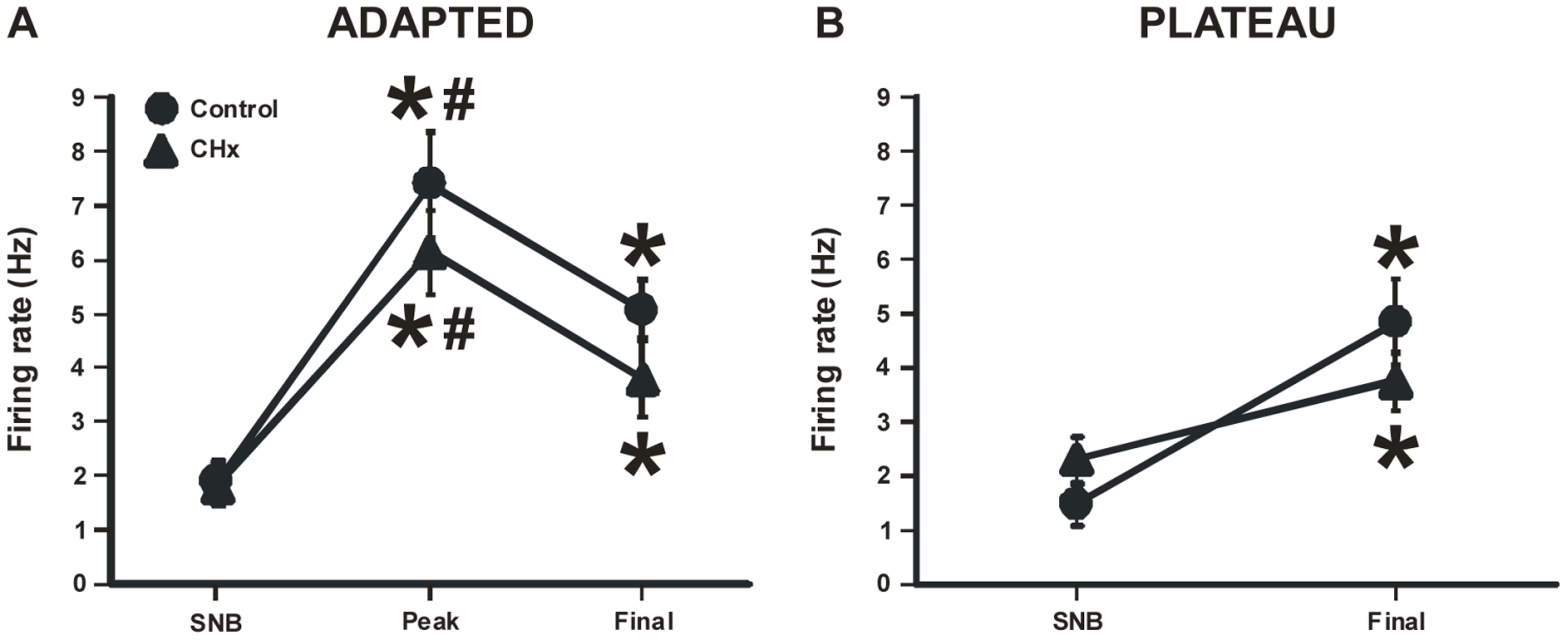
Average adapted and plateau basal firing rate response to substance P. The average adapted (A) and plateau (B) basal firing rate response to substance P (SP) for SC neurons from both control (filled circles) and CHx rats (filled triangles). (A) The first point represents firing rate in the presence of synaptic blockade (SNB) solution, the second point represents the peak response to SP, and the last point represents the final response to SP. Average adapted basal firing rate increased in response to SP to a peak and then adapted to a steady final value for SC neurons from control rats. The firing rate responses to SP of SC neurons did not differ between control and CHx rats for the adapted response. (B) The first point represents firing rate in the presence of SNB solution and the second point represents the plateau or final response to SP. The firing rate responses to SP of SC neurons did not differ between control and CHx rats for the plateau response. *indicates that firing rate is significantly different than the firing rate in SNB. ^#^indicates that the firing rate at peak is significantly different than the final firing rate. In all cases, points represent the mean firing rate ± S.E.M.

Adapted and plateau firing rate responses to substance P were next compared in SC neurons from control and CHx rats. In control rats, 64% of SC neurons (14/22) showed adapted responses and 36% (8/22) showed plateau firing rate responses to substance P. These percentages shifted slightly, but not significantly (P = 0.145) in CHx rats, where 43% of SC neurons (10/23) showed adapted responses and 57% (13/23) showed plateau firing rate responses to substance P. SC neurons from both control and CHx rats showed a similar adapted firing rate response to substance P, which involved an initial significant (P<0.001) peak increase in firing rate that then adapted to a significantly (P<0.001) smaller firing rate (Fig. 4A). In summary, the adapted firing rate response was still significantly larger than the initial firing rate for SC neurons from both control and CHx rats, but firing rates of SC neurons at each time-point did not differ when comparing control and CHx rats.

SC neurons that exhibited a plateau response to substance P had firing rates that slowly increased in response to substance P until they plateaued (Fig. 4B). Substance P significantly increased firing rate in SC neurons from both control (P<0.001) and CHx (P<0.05) rats (Fig. 4B). Similar to the adapted response to substance P, we observed that firing rates of SC neurons that had a plateau response did not differ when comparing control and CHx rats. Note that a stable firing rate of 4 Hz was reached in the presence of substance P for all neurons regardless of the type of response (adapted or plateau) (Fig. 4).

### Effect of Substance P on the Chemosensitive Response of SC Neurons from both Control and CHx Rats

**Figure 5:**
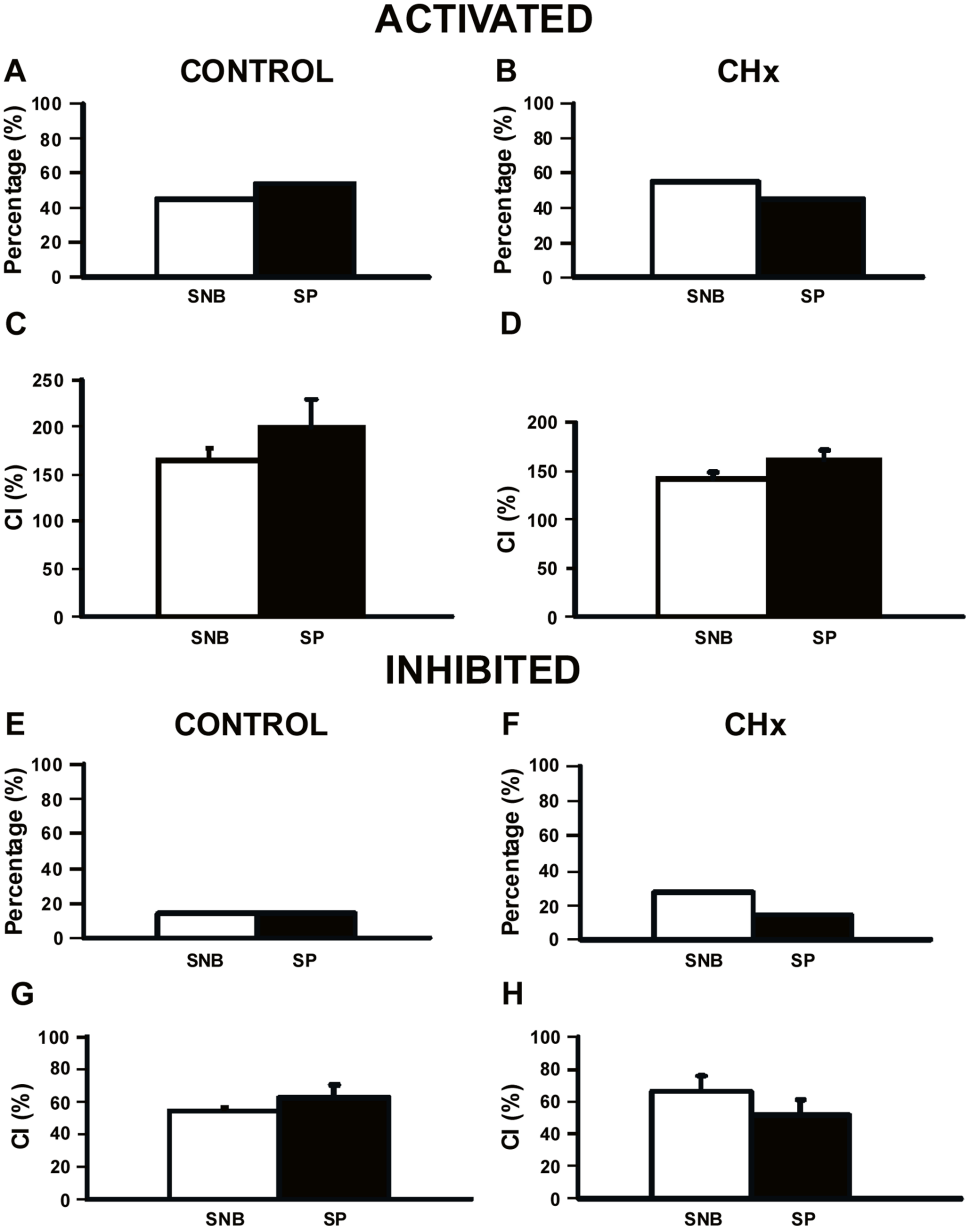
Chemosensitivity response of SC neurons to substance P. Percentages of SC neurons that were activated (A and B) or inhibited (E and F) by hypercapnic acidosis in the absence (SNB) or presence of substance P (SP) from control rats (N = 10 and N = 12 in the absence and presence of SP for CO_2_-activated SC neurons, A; N = 3 in the absence and presence of SP for CO_2_-inhibited SC neurons, E) and from CHx rats (N = 13 and N = 10 in the absence and presence of SP for CO_2_-activated SC neurons, B; N = 6 and N = 3 in the absence and presence of SP for CO_2_-inhibited SC neurons, F). Chemosensitivity indices (CI) of SC neurons (same neurons as in A, B, E and F) that were CO_2_-activated (C and D) or CO_2_-inhibited (G and H) by hypercapnic acidosis in the absence or presence of SP from control rats (C and G) and CHx rats (D and H). In all cases, white bars represent synaptic blockade (SNB) solution and black bars represent SP. (A-D) The percentage and CI of SC neurons that were activated by hypercapnic acidosis in the absence and presence of SP from control and CHx rats, where the percentage and CI were not affected by SP. (E-H) The percentage and CI of SC neurons from control and CHx rats that were inhibited by hypercapnic acidosis in the absence and presence of SP, where the percentage and CI were not affected by SP. The height of each bar represents either the percentage CO_2_-activated (A and B) or CO_2_-inhibited (E and F) or the mean CI for that group (C, D, G and H) ± S.E.M.

One of the main goals of this study was to examine the effect of substance P on the chemosensitive response of SC neurons from both control and CHx rats. We quantified the chemosensitive response to substance P by looking at the percentage of neurons that respond and the magnitude of the response, or the CI, in neurons isolated from their synaptic inputs in SNB medium. As shown previously [35], chronic hypoxia resulted in no change of the percentage of hypercapnia-activated SC neurons (Figs. 5A, B). Substance P did not change the percentage of CO_2_-activated SC neurons from either control rats (Fig. 5A) or from CHx rats (Fig. 5B). Similarly, the CI of CO_2_-activated SC neurons from control rats (Fig. 5C) and from CHx rats (Fig. 5D) was unchanged by substance P. Thus, the addition of substance P did not affect the response of SC neurons activated by hypercapnic acidosis in control or CHx rats.

We saw very few hypercapnia-inhibited SC neurons in the absence of substance P in both control and CHx rats (Figs. 5E, F), although there was a trend towards an increase in inhibited SC neurons in CHx rats as shown before [35]. Substance P did not affect this relationship (Figs. 5E, F). CI of CO_2_-inhibited SC neurons was unaffected by substance P in control rats (Fig. 5G) and CHx rats (Fig. 5H). Together, our data suggest that substance P modulates basal firing rate, but has no effect on the chemosensitive response of SC neurons from either control or CHx rats (Fig. 5).

### Effect of NK1 Receptor Antagonist (L-703,606) on Basal Firing Rate of SC Neurons from both Control and CHx Rats

In order to verify that substance P was not working through a receptor other than the NK1 receptor, we used an NK1 receptor antagonist, L-703,606, to block the effect of substance P on SC neurons from both control and CHx rats. We first wanted to check the specificity of L-703,606 to ensure that it was blocking the substance P response. L-703,606 significantly inhibited the substance P-induced increased basal firing rate response (by ∼70–90%) (expressed as a substance P-induced change in firing rate from baseline) of SC neurons from both control (4.71±0.59 Hz in the presence of SNB ± substance P to 0.61±0.42 Hz in the presence of SNB+L-703,606 and substance P; P<0.0001) and CHx rats (2.67±0.59 Hz in the presence of SNB+substance P to 0.85±0.66 Hz in the presence of SNB+L-703,606 and substance P; P = 0.0431; data not shown). Thus, it appears that substance P is predominately working through the NK1 receptor.

### Effect of NK1 Receptor Antagonist (L-703,606) on the Chemosensitive Response of SC Neurons from both Control and CHx Rats

**Figure 6:**
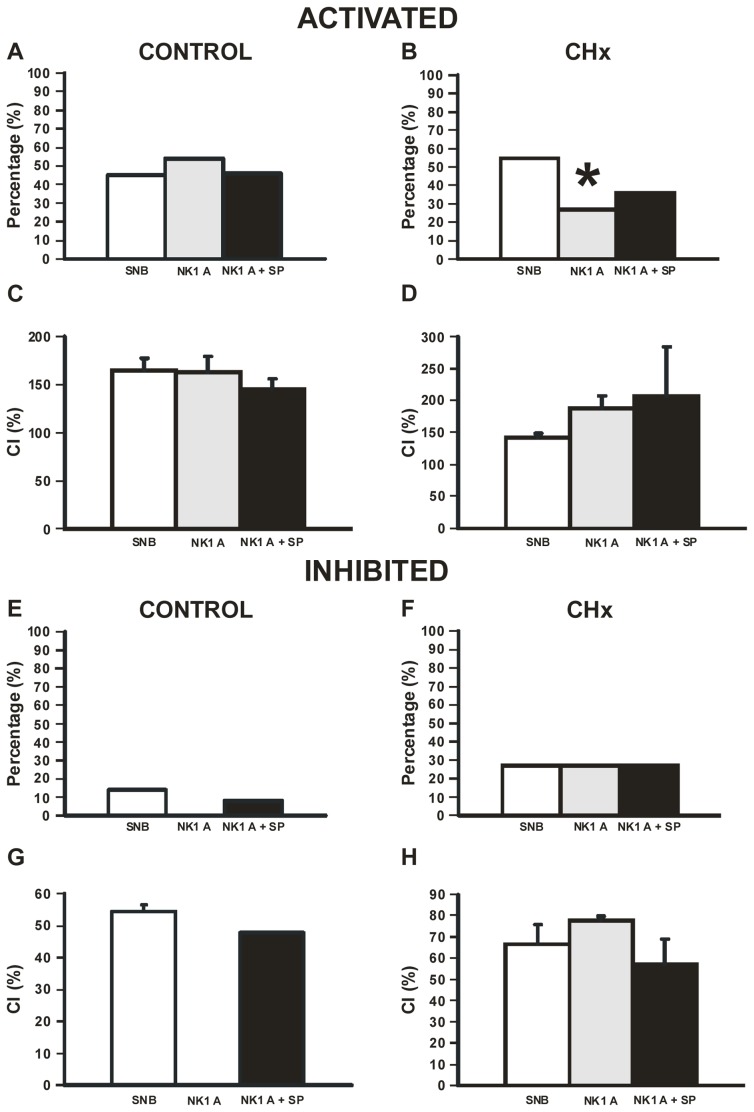
Chemosensitivity response of SC neurons following NK1 receptor inhibition. Percentages (A, B, E, and F) and chemosensitivity indices (CI; C, D, G and H) for SC neurons that were activated (A-D) or inhibited (E-H) by hypercapnic acidosis in the presence of SNB (white bars), NK1 receptor antagonist (NK1 A; L-703,606; gray bars), and in the presence of NK1 A and substance P (NK1 A+SP; black bars) from control (A, C, E, and G) and CHx rats (B, D, F and H). (A and B) Percentages of SC neurons from control and CHx rats that were activated by hypercapnic acidosis in SNB (N = 10 for control; N = 13 for CHx), NK1 A (N = 7 for control; N = 3 for CHx) or NK1 A+SP (N = 6 for control; N = 4 for CHx), where the percentage activated by hypercapnic acidosis was only decreased by NK1 A alone (P<0.05 represented by *) in SC neurons from CHx rats. (C and D) CI of SC neurons from control and CHx rats that were activated by hypercapnic acidosis was not affected when comparing SNB, NK1 A or NK1 A+SP. (E-H) Percentages and CIs of SC neurons from control and CHx rats that were inhibited by hypercapnic acidosis were not affected when comparing SNB (N = 3 for control; N = 6 for CHx), NK1 A (N = 0 for control; N = 3 for CHx) or NK1 A+SP (N = 1 for control; N = 3 for CHx). Note, there is no error bar for CI for the NK1 A+SP experiment since both neurons had the same CI (G). The height of each bar represents either the percentage CO_2_-activated or CO_2_-inhibited (A, B, E, and F) or the mean CI for that group (C, D, G and H) ± S.E.M.

We then quantified the chemosensitive responses of SC neurons from control (n = 13) and CHx rats (n = 11) in the presence of SNB and L-703,606 or in the presence of SNB, L-703,606 plus substance P. These responses were compared to those of SC neurons in SNB only (Fig. 5). We quantified the percentage of neurons that responded to hypercapnia and their CI. In the presence of SNB and L-703,606 (N = 7) or SNB, L-703,606 and substance P (N = 6), the percentage of CO_2_-activated SC neurons and their CI were the same in control rats (Figs. 6A, C). For CO_2_-activated SC neurons from CHx rats, L-703,606 significantly (P<0.05) reduced the percentage of CO_2_-activated neurons without affecting CI (Figs. 6B, D). When substance P was combined with L-703,606 in CO_2_-activated SC neurons, no significant differences were observed in percentage or CI (Figs. 6B, D). In the presence of SNB and L-703,606 or SNB, L-703,606 and substance P, the percentage of CO_2_-inhibited SC neurons and their CI did not change in control (Figs. 6E, G) or CHx (Figs. 6F, H) rats. Thus, L-703,606 blunts the firing rate response of SC neurons to substance P, but when this antagonist is applied alone or along with substance P, the firing rate response to hypercapnic acidosis of SC neurons from both control and CHx rats is largely unchanged. This suggests that basal substance P release may be necessary for some SC neurons to exhibit CO_2_ activation, but endogenous or exogenous substance P has no effect on the magnitude of the firing rate response to hypercapnic acidosis in this population of SC neurons from either control or CHx rats.

### Effect of Substance P and/or L-703,606 on Basal pH_i_ of SC Neurons from both Control and CHx Rats

In addition to recording basal firing rate responses to substance P, we also simultaneously measured the basal pH_i_ response to substance P and/or L-703,606. Substance P did not induce any effects on steady state pH_i_ within any of the three groups (CO_2_-activated, CO_2_-inhibited or non-chemosensitive SC neurons; data not shown) from control or CHx rats, so we decided to combine the effect of substance P on pH_i_ of all SC neurons for both groups of rats. After grouping all SC neurons together, substance P induced a small significant acidification (7.24±0.01 in the absence of substance _P_ and 7.21±0.02 in the presence of substance P; N = 22; P<0.05) in SC neurons from control rats and CHx rats (7.23±0.02 in the absence of substance P and 7.17±0.03 in the presence of substance P; N = 23; P<0.001). There were no significant differences in the effect of substance P on pH_i_ found between control and CHx rats. This small substance P-induced acidification may be related to the substance P-induced increased firing rate, which has been shown in other neurons to induce a small acidification [39]. We also measured the effect of L-703,606 on steady state pH_i_ (first in the presence of SNB+L-703,606 and then in the presence of SNB+L-703,606+ substance P) and found that in the presence of antagonist substance P did not significantly change pH_i_ from either control or CHx rats.

### Effect of Substance P on the pH_i_ Response to Acute Hypercapnia of SC Neurons from both Control and CHx Rats

Finally, we measured the effect of substance P on pH_i_ during acute hypercapnia as we did for examining the effect of substance P on steady state pH_i_, in that we grouped all SC neurons together from control rats and we grouped all SC neurons together from CHx rats. The magnitude of acidification induced in response to acute hypercapnia in the presence of substance P (0.24±0.01 pH unit) was significantly larger than the change induced in the absence of substance P (0.19±0.01 pH unit; P<0.01) for SC neurons from control rats, although this difference is rather small. On the other hand, in SC neurons from CHx rats substance P did not affect the pH_i_ response to acute hypercapnia (0.23±0.02 pH unit in the absence of substance P to 0.27±±0.02 pH unit in the presence of substance P). Thus, substance P does not appear to have much of an effect on hypercapnia-induced acidification in adult rat SC neurons.

## Discussion

Substance P release from peripheral afferents increases in response to hypoxia [29] and these peripheral afferents synapse on caudal NTS neurons, where NK1 receptors are known to be expressed [25], [27], [34]. It has been suggested that NK1 receptors can be used for markers of neurons that are involved in breathing, including ones participating in rhythm generation and central chemosensitivity [25]. However, neither the cellular response of SC neurons to substance P nor the chemosensitive response of these neurons have been studied in control rats *vs*. rats adapted to chronic hypoxia. Thus, the main question of this study was to examine the chemosensitive response of SC neurons in the presence of substance P. The main findings of this study include: 1) substance P increases the basal firing rate of all SC neurons in control rats, but only SC neurons activated by hypercapnia in CHx rats; 2) substance P has no effect on the firing rate response to acute hypercapnia of SC neurons from either control or CHx rats; and 3) when NK1 receptors are blocked we see no effect on the firing rate response of SC neurons to acute hypercapnia. Thus, substance P has a role in modulating basal firing rate, but does not appear to be involved in the firing rate response to acute hypercapnia in SC neurons from adult rats.

### Substance P Effect on Basal Firing Rate: A Potential Role for Substance P in Ventilation

There is evidence that multiple sources of substance P can affect NTS neurons including release within the NTS [13], [27] as well as substance P release from peripheral afferents arising from the carotid body that synapse in the NTS [17]. Previously, it was shown that when substance P was applied to the NTS, respiratory frequency, tidal volume and therefore minute ventilation increased [5], [6], [7], [8], [9]. Thus, we hypothesized that substance P would have an excitatory effect on NTS neurons *in vitro*. Our data are consistent with these findings in that the increase in breathing that occurs after substance P is injected could be the result of substance P increasing the firing rate of SC neurons, as we observed here (Fig. 3).

SC neurons have two different responses to substance P (adapted and plateau) but, regardless of the type of response, firing rate seemed to stabilize at around 4–5 Hz during prolonged exposure to substance P of SC neurons from both control and CHx rats (Fig. 4). Substance P caused a significant increase in firing rate of all three types of SC neurons (CO_2_-activated, non-chemosensitive and CO_2_-inhibited) from control rats, but only increased firing rate of CO_2_-activated SC neurons from CHx rats (Fig. 3). These findings suggest that that expression of NK1 receptors cannot be used as a marker of chemosensitive neurons [25], at least for SC neurons. Moreover, we are not sure why there are different patterns of responses to substance P or why the firing rate ultimately stabilizes at 4–5 Hz, but this represents a substantial increase in firing relative to basal firing rate. We speculate that the two patterns are seen because either: 1) two different substance P receptors are present, one that is susceptible to desensitization and one that is not; and/or 2) the receptor that is susceptible to desensitization is modulated by chronic hypoxia exposure. The latter speculation is consistent with our data since we observed a slight shift in the number of neurons that had an adapted response (10/23 for CHx rats and 14/22 for controls) and plateau response (13/23 for CHx rats and 8/22 for controls) to substance P after chronic hypoxia. Moreover, chronic hypoxia could increase release of substance P, and our data suggests that any central effect of substance P in SC neurons from CHx rats is most likely a pre-synaptic (*i.e.* increased release) rather than a post-synaptic (*i.e.* increased responsiveness to substance P) effect.

### Substance P Effect on the Hypercapnic Ventilatory Response

Substance P is believed to affect breathing, although the specific role it plays is controversial. In preparations in which various chemosensitive regions were exposed to substance P, respiratory output increased [5], [6], [7], [8], [9], [47], [48], [49], suggesting that NK1 receptor expressing neurons increase basal firing rate of neurons within these regions (consistent with the data observed in this study as discussed above). However, the role for NK1 receptor-expressing neurons in the hypercapnic ventilatory response and/or substance P playing a modulatory role is debatable. Following microinjection of saporin conjugated to substance P in the RTN and the medullary raphe, the hypercapnic ventilatory response was decreased, which suggests that neurons expressing NK1 receptors may play a role in modulating central chemosensitivity [10], [11], [12], [50]. In contrast, the hypercapnic ventilatory response was unaffected in control and CHx rats following microinjection of saporin conjugated to substance P in the caudal NTS [51]. Moreover, hypercapnia-induced depolarization was not inhibited in RTN neurons when a substance P receptor antagonist was injected into the RTN, suggesting that substance P *per se* does not have a modulatory role in the hypercapnic ventilatory response [52]. However, following injection of an NK1 receptor inhibitor into the VLM, the hypercapnic ventilatory response did decrease but when injected into the dorsal medulla (including the NTS), it had no effect [9]. Further, substance P knockout mice had the same frequency response to inspired CO_2_ as control mice, suggesting that substance P does not play a major modulatory role in respiratory control [53]. Overall, these data argue that substance P does not appear to play a major role in the hypercapnic ventilatory response and our data are consistent with this conclusion since the firing rate responses to acute hypercapnia of SC neurons from control and CHx rats were not affected by substance P application or NK1 receptor inhibition.

Although substance P does not appear to have a direct role in the chemosensitive response to hypercapnia of caudal NTS neurons, it does not mean that substance P does not play a role in modulating the hypercapnic ventilatory response. The ventilatory response to hypercapnia is likely to be mediated by a complex network involving several brainstem regions that are highly inter-connected [54]. For instance, neurons of the caudal NTS have been shown to project to other chemosensitive regions, including the rostral ventrolateral medulla [55], the Kölliker-Fuse [56], the RTN [57], and the locus coeruleus [58], [59]. Although we do not know the details of the communication between these regions, our findings that substance P stimulates the firing rate of virtually all caudal NTS neurons under normocapnic conditions (Figs. 1, 2 and 3), suggest that substance P activation of these caudal NTS neurons can modulate the activity and responsiveness of neurons from numerous other chemosensitive areas. Thus, the effect of substance P on the basal firing rate of caudal NTS neurons could alter the network responsiveness to hypercapnia leading to a modulatory role for substance P in the hypercapnic ventilatory response that involves caudal NTS neurons. Further, since substance P only significantly stimulates the basal firing rate from chemosensitive neurons in rats adapted to chronic hypoxia, it is likely that chronic hypoxia would alter the modulatory role for substance P in the hypercapnic ventilatory response that is mediated by caudal NTS neurons.

### Substance P Effect on the Hypoxic Ventilatory Response

Substance P may affect the hypoxic ventilatory response. The hypoxic ventilatory response was decreased following injection of an NK1 receptor inhibitor into the VLM or the ventricles of the brain [13]. Interestingly, NK1 receptor knockout adult mice have a reduced ventilatory response to hypoxia [60]. These reductions may involve changes within the NTS. It is known that peripheral afferents arising from the carotid body synapse on NTS neurons [14], [15], [16], [17], [18], [19]. Neurons within regions playing a role in breathing also can respond to changes in oxygen, including the neurons from the NTS [61], [62], [63], [64], [65], [66], [67]. Here, we observed a lack of an effect of substance P on the response of SC neurons to hypercapnia. However, this does not rule out the possibility that substance P may play a role in the response of these neurons to acute hypoxia, especially on SC neurons from CHx rats.

## Conclusions

Our study is one of the first to look for a possible role of substance P in modulating CO_2_-sensitive neurons *per se*. We designed our experiments to employ the ability of hypoxia to modulate substance P levels and we studied the NTS because it is known to have altered substance P levels and exhibit plasticity in response to hypoxia [29], [30], . The findings reported in this study represent the first time substance P has been shown to directly modulate basal firing rate of SC neurons, but have no effect on the firing rate response of SC neurons to acute exposures to hypercapnic acidosis. Thus, our data may explain how basal ventilation is increased after focal injection of substance P. However, we find no direct evidence that substance P is playing a major role in modulating the hypercapnic ventilatory response, consistent with previous studies [9], [52], [53]. Our study cannot rule out a role for substance P in modulating the response to hypercapnia of other chemosensitive neurons, especially in the RTN or rostral VLM, which would be an interesting future study. It does appear that, like in studies of other NTS neurons following exposure to chronic intermittent hypoxia [34], chemosensitive NTS neurons also see a potential decrease in NK1 receptors following chronic sustained hypoxia and possibly have less activation by substance P. Lastly, our study also cannot rule out a role for substance P in modulating the hypoxic ventilatory response. *In conclusion*, our data suggest that substance P in the SC can modulate basal respiratory drive.
